# Supplementary material for: Ultrasound-Assisted Mineralization of 2,4-Dinitrotoluene in Industrial Wastewater Using Persulfate Coupled with Semiconductors
Source: Molecules. 2023 May 25;28(11):4351. doi: 10.3390/molecules28114351 (PMC10254761; doi:10.3390/molecules28114351)
Supplement: Supplementary file 1 [file molecules-28-04351-s001.zip › molecules-2372276-supplementary.pdf]

Supplementary Figure

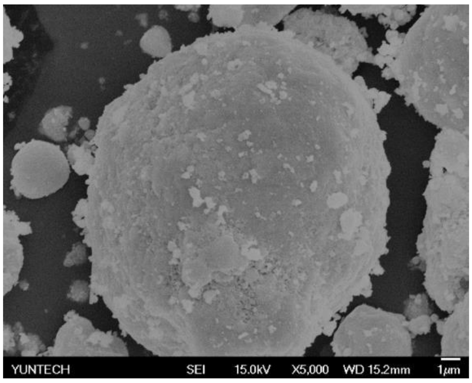

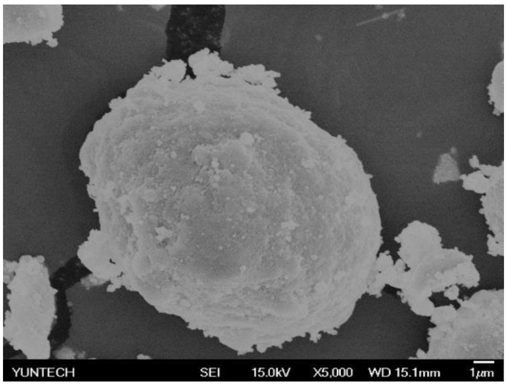

(a) Ag(1wt%)/NiO (b) Ag(2wt%)/NiO

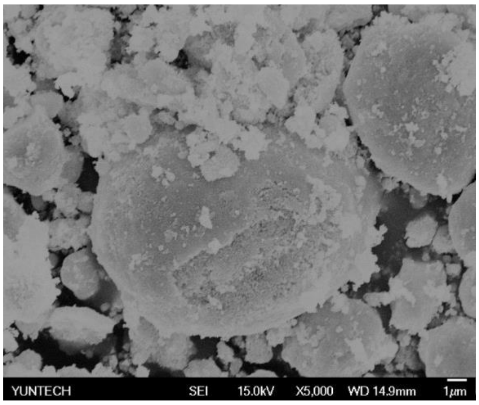

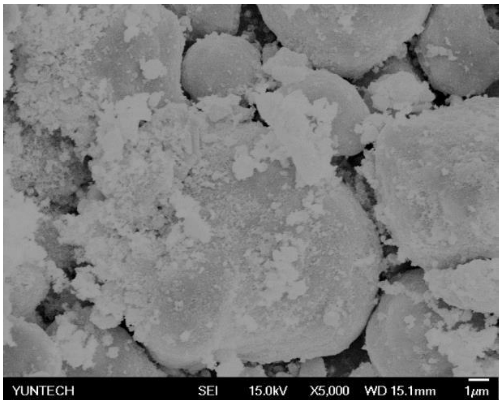

(c) Ag(3wt%)/NiO (d) Ag(4wt%)/NiO

Figure S1. FE-SEM images of the (a) Ag(1wt%)/NiO, (b) Ag(2wt%)/NiO, (c) Ag(3wt%)/NiO and (d) Ag(4wt%)/NiO semiconductors.

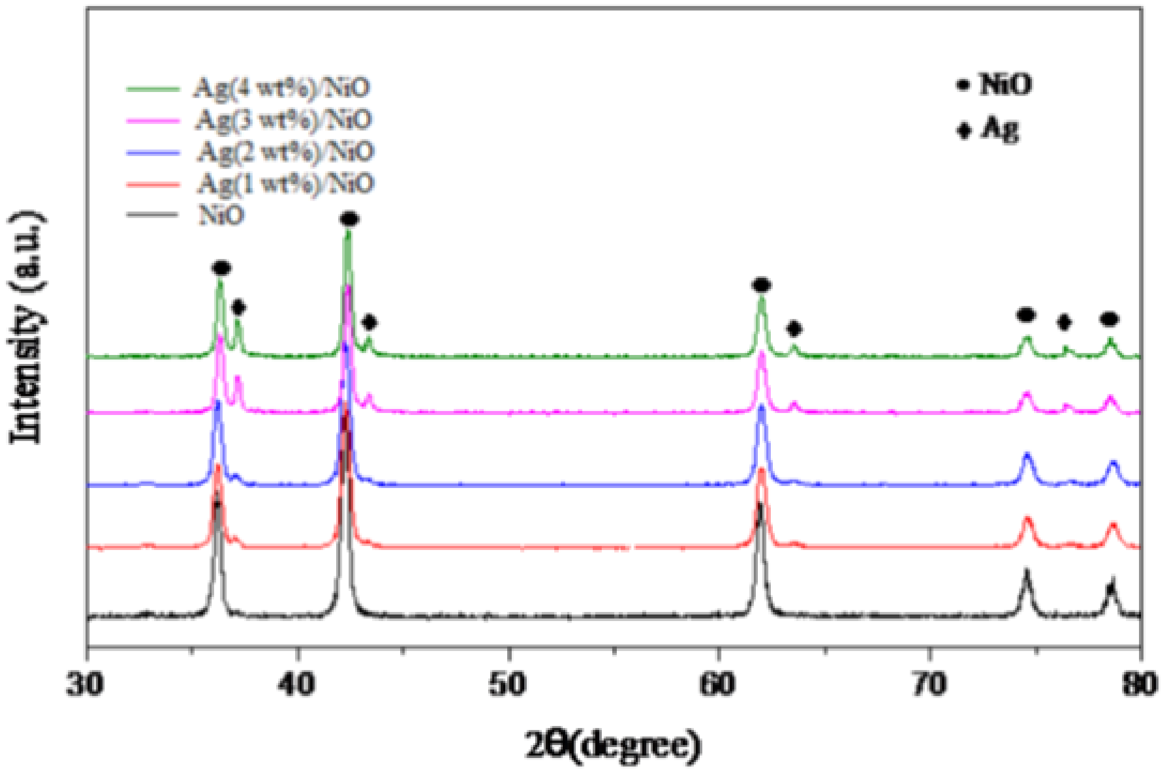

Figure S2. X-ray diffraction patterns of Ag(1wt%)/NiO, Ag(2wt%)/NiO, Ag(3wt%)/NiO and Ag(4wt%)/NiO semiconductors.
